# Novel Endosymbionts in Rhizarian Amoebae Imply Universal Infection of Unrelated Free-Living Amoebae by Legionellales

**DOI:** 10.3389/fcimb.2021.642216

**Published:** 2021-03-08

**Authors:** Marcel Dominik Solbach, Michael Bonkowski, Kenneth Dumack

**Affiliations:** Terrestrial Ecology Group, Institute of Zoology, University of Cologne, Cologne, Germany

**Keywords:** *Legionella*, endosymbiotic bacteria, pneumonia, free-living amoebae, biofilms, Coxiellaceae, Cercozoa, gnotobiotic protist culture

## Abstract

Legionellales-infected water is a frequent cause of local outbreaks of Legionnaires’ disease and Pontiac fever. Decontaminations are difficult because Legionellales reproduce in eukaryotic microorganisms (protists). Most often, Legionellales have been isolated from amoebae; however, the culture-based sampling methods are taxonomically biased. Sequencing studies show that amoebae in the cercozoan class Thecofilosea are dominant in soils and wastewater treatment plants, prompting us to screen their capability to serve as potential hosts of endosymbiotic bacteria. Environmental isolates of Thecofilosea contained a surprising richness of endosymbiotic Legionellales, including *Legionella*. Considering the widespread dispersal of Legionellales in apparently unrelated amoeboid protist taxa, it appears that the morphotype and not the evolutionary origin of amoebae determines their suitability as hosts for Legionellales. We further provide a protocol for gnotobiotic cultivation of Legionellales and their respective hosts, facilitating future genomic and transcriptomic research of host–symbiont relationships.

## Introduction

Legionellales are common intracellular bacteria in a variety of eukaryotes (Boamah et al., 2017; Duron et al., 2018). Their function, *i.e.* whether they act as exploitatory parasites or mutualistic endosymbionts, in most hosts is unexplored, but infections of humans often cause serious diseases including Legionnaires’ disease, Pontiac fever, or Q fever (Cunha et al., 2016; Beauté and Robesyn, 2017; Eldin et al., 2017). Humans are usually infected through inhalation of contaminated aerosols or aspiration of contaminated water (Shelton et al., 2010). The symptoms vary from common cold-like symptoms to fatal pneumonia. Confirmed cases of Legionnaires’ disease range in several thousand cases per 100,000 population per year, but the exact number of cases per year is difficult to estimate since official bodies assume widespread underdiagnosis (Beauté and Robesyn, 2017; Shah et al., 2019). The European Center for Disease Prevention and Control reports a fourfold increased notification rate of Legionnaires’ disease in the EU/EEA between the years 1995 to 2015 (Beauté and Robesyn, 2017). Due to a 5.5-fold increase of cases between the years 2000 and 2017 in the US, the U.S. Center for Disease Control and Prevention classifies Legionnaires’ disease as “on the rise” (The Centers for Disease Control and Prevention (CDC)). Since Legionellales are common in the environment, an increased local abundance may thus quickly lead to fatal local disease outbreaks (Morton et al., 1986; van Loenhout et al., 2012). Under natural conditions, Legionellales bacteria rely on a eukaryotic host to proliferate (Fields et al., 2002; Declerck et al., 2009; Declerck, 2010). Accordingly, fluctuations in the abundance of Legionellales bacteria are strongly linked to fluctuations of the abundance of their hosts in the environment (Scheikl et al., 2014; Amaro et al., 2015). However, surprisingly little is still known on their host ranges. As parasites in humans and animals, Legionellales infect the amoeba-like alveolar macrophages (Escoll et al., 2013; Duron et al., 2018). In the environment, Legionellales were found to reside and reproduce in protists, strikingly often in various kinds of amoebae (Fields et al., 2002; Duron et al., 2018).

It is hence commonly stated that Legionellales proliferate within amoebae; this statement, however, is not trivial. Amoebae taxonomy has been in permanent change during the last decades. Initially, the lack of clear morphological characters in amoeboid microorganisms hampered their classification. With the application of molecular tools, the eukaryotic tree of life was reshaped and ‘amoeba’-like morphology turned out as a polyphyletic trait. It is now widely accepted that the term ‘amoebae’ describes an assemblage of morphologically similar, but only very distantly related microeukaryotes (Adl et al., 2019; Burki et al., 2019). By far most recognized hosts of Legionellales are amoebae in the higher-order Amorphea, including host genera like *Acanthamoeba*, *Vermamoeba* (former *Hartmannella vermiformis*)*, Arcella, Vannella*, *Cochliopodium*, and *Nuclearia* (Santos et al., 2003; Dirren and Posch, 2016; Mehari et al., 2016; Tsao et al., 2017; Gomaa et al., 2018; Galindo et al., 2019). Non-amorphean amoeba hosts seem rare, although Legionellales were also detected in the distantly related amoeba genus *Naegleria* (Excavata; (Delafont et al., 2013)).

The Rhizaria are rather unrelated to the previously mentioned taxa, but independently evolved a huge variety of amoeboid taxa. The Cercozoa (Rhizaria) are particularly species-rich and dominant in terrestrial and aquatic habitats (Lentendu et al., 2014; Grossmann et al., 2016). Despite their commonness, not a single amoeba belonging to the Cercozoa has yet been investigated for putative endosymbiotic relationships with bacteria like the Legionellales. Hereinafter, we use the term “endosymbiosis” in its most general definition encompassing the broad range of intracellular associations, ranging from mutualism to parasitism.

We aimed to investigate whether Rhizaria may function as hosts for Legionellales; as a starting point, we chose to screen the amoeboid Thecofilosea (Cercozoa, Rhizaria) since molecular investigations indicate an occasional dominance of these small and inconspicuous microorganisms in soils, wastewater treatment plants, and water filters (Matsunaga et al., 2014; Remmas et al., 2016a; Remmas et al., 2016b; Seppey et al., 2017; Fernandez, 2019; Öztoprak et al., 2020).

We screened 13 strains of Thecofilosea for potential bacterial endosymbionts and found a surprising taxonomic richness of endosymbiotic Legionellales with eight of 13 screened amoebae accommodating Legionellales bacteria and only one of the 13 strains accommodating a non-Legionellales endosymbiotic bacterium. Four different species of Legionellales bacteria including species of the potentially pathogenic genus *Legionella* and two novel Legionellales genera were detected. The incongruence of the phylogenies of the amoeboid hosts and their bacterial endosymbionts suggests a promiscuous distribution of Legionellales in amoeboid hosts of wide evolutionary origin. The amoeboid lifestyle appears as the only common denominator in this relationship.

## Material and Methods

### Sampling and Cultivation

Samples were taken in several different habitats including soil, freshwater, and plant leaves. Leaves and soil samples were submerged in Waris-H+Si (Mcfadden and Melkonian, 1986) and incubated for at least one day before analyses to enable microscopic observation of attached protists. The samples were screened weekly for up to three weeks after collection for Thecofilosea-like cells with a Nikon Eclipse TS100 inverted microscope (up to 400× magnification, phase contrast). Single cells were picked with a glass micropipette and transferred into a well of a 24-well plate (Sarstedt, Nümbrecht, Germany), containing 2 ml of the respective culture medium and food source. Subculturing was repeated until a monoclonal culture free of other eukaryotes was obtained. Depending on the species, strains were cultured in either Waris-H+Si (Mcfadden and Melkonian, 1986), WC-Medium (Guillard and Lorenzen, 1972) or Wheat Grass (WG)-Medium (Bonkowski, 2019). For a list of respective culture media and food sources refer to **Supplementary Table S1**.

To obtain gnotobiotic *Fisculla* strains, wells of 96-well plates were filled with 50 µl sterile Waris-H+Si medium and axenic *Saccharomyces cerevisiae*. Single *Fisculla* cells were sorted by size (approximately 10–20 µm diameter) *via* flow cytometry (BD FACSVantage, Becton, Dickinson and Company, Franklin Lakes, New Jersey). Plates were incubated for approximately five days at room temperature before further analysis to allow cultures to grow. The 96-well plates were then screened for wells free of environmental bacteria and containing only cells of *Fisculla* spp., and the remaining *S. cerevisiae*. Respective wells were used for subsequent inoculation of 1.25% Waris-H+Si agar. Waris-H+Si agar (1.25%) was obtained by autoclaving 2.5 g of agar (Sigma-Aldrich, Munich, Germany) in 200 ml Waris-H+Si. Plates were poured under a sterile bench. Once the agar dried, axenic *S. cerevisiae* cells were distributed with a cell scraper. Plates were stored at approximately 14–16°C in the dark.

### Sequencing and Cloning

To reduce the number of environmental bacteria (if present) before amplification and sequencing of bacterial DNA, amoeba cells were preferably separated from environmental bacteria. Single *Fisculla* cells were sorted *via* flow cytometry to Waris-H+Si containing axenic *S. cerevisiae* as food organism and collected when all food cells were consumed (approx. after one week of incubation). Three hundred *Rhogostoma* cells of each strain were sorted *via* flow cytometry and collected without further incubation for DNA extraction. The Thecofilosea sp. CCAP 1943/6 cells were too large and too low in density to be sorted and thus were filtered (filter pore size: 30 µm). The filter was given in medium and directly afterwards 150–200 cells were collected with a glass micropipette. DNA extraction of the collected cells was performed with a DNEasy Blood and Tissue kit (Qiagen, Venlo, Netherlands). Extracted DNA was stored at −21°C.

DNA amplification of the hypervariable region V4 of the SSU rDNA of the amoeba strains was performed. Preferably single individuals with approximately 1 µl of medium were transferred into PCR-tubes, containing 17 µl PCR mixture. The mixture included 1.7 µl 10 µM forward and 1.7 µl 10 µM reverse primer, 0.34 µl 10 mM dNTPs, 1.7 µl Thermo Scientific Dream Taq Green Buffer, 0.17 µl DreamTaq polymerase (Thermo Fisher Scientific, Dreieich, Germany), and 11.4 µl water. Sequences of the SSU rDNA were obtained in two consecutive steps. First, a sequence with a length of approximately 347 bases was amplified with the Cercozoa-specific primers S616F_Cercomix and S963R_Cerco (Fiore-Donno et al., 2018). In the second step, semi-nested reamplifications were performed with a PCR mixture containing the same components as above and the primer pairs S947R_Cerco and S616F_Cercomix (Fiore-Donno et al., 2018). Two µl of the first PCR product was used as template. The following PCR conditions were used: initial denaturation at 95°C for 2 min, 30 cycles (denaturation at 95°C for 30 s, annealing at 52°C for 30 s, elongation at 72°C for 30 s), terminal extension at 72°C for 5 min, and cooling at 4°C.

DNA amplification of the 16S rDNA of bacteria was performed with 2 µl of extracted DNA as template with a PCR mixture containing the same components as above and the bacterial primers 27F and 1492R (Weisburg et al., 1991; Jiang et al., 2006). The following PCR conditions were used: initial denaturation at 95°C for 2 min, 30 cycles (denaturation at 95°C for 30 s, annealing at 48°C for 30 s, elongation at 72°C for 90 s), terminal extension at 72°C for 5 min, and cooling at 4°C. PCR products were purified by adding 0.15 µl of Exonuclease, 0.9 µl FastAP and 1.95 µl water to 8 µl PCR product, then heating for 30 min at 37°C, and subsequently for 20 min at 85°C. The Big dye Terminator Cycle sequencing Kit (Thermo Fisher Scientific, Dreieich, Germany) and an ABI PRISM automatic sequencer were used for sequencing.

When direct sequencing of the bacteria did not result in a distinct sequence, 100 µl PCR products were subjected to cloning. Two µl of extracted DNA was used as template. PCR products were purified using the GeneJET PCR Purification Kit (Thermo Fisher Scientific, Dreieich, Germany) and eluted in 20 µl elution buffer. Ligation and transformation were performed according to the manufacturer’s instructions using the pGEM^®^-T Easy Vector System with JM109 Competent Cells (Promega GmbH, Mannheim, Germany), with the following changes to the protocol: Ligation was conducted overnight at 5°C using 2.5 µl 2× Rapid Ligation Buffer, 0.5 µl pGEM^®^-T Easy Vector (50 ng), 0.5 µl T4 DNA Ligase, 0.5 µl H_2_O, and 1 µl of the purified PCR product. In step 6 of the instructions, only 250 µl of SOC medium was added to the ligation reactions. LB agar plates were obtained by dissolving 9.6 g of LB-Agar—Powder according to Miller (AppliChem, Darmstadt, Germany) in 300 ml water. The mixture was autoclaved, then 175 µl Ampicillin (100 mg/ml), 300 µl 100 mM IPTG (Thermo Fisher Scientific, Dreieich, Germany), and 600 µl X-Gal Solution, ready-to-use (Thermo Fisher Scientific, Dreieich, Germany) were added to the liquid agar once it cooled down to approximately 50°C. Plates were poured under a sterile bench. For every strain 50 µl and 150 µl of the transformation cultures were plated onto duplicate LB agar plates. Plates with transformation cultures were incubated overnight at 37°C. White colonies were picked with a sterile toothpick and directly added to 10 µl PCR mix. The mixture included 0.3 µl 10 µM forward and 0.3 µl 10 µM reverse primer, 0.3 µl 10 mM dNTPs, 1 µl Thermo Scientific Dream Taq Green Buffer, 0.1 µl DreamTaq polymerase, and 8 µl water. DNA amplification of the colonies was performed using the primers M13-40 and M13R. The following PCR conditions were used: initial denaturation at 95°C for 2 min, 30 cycles (denaturation at 95°C for 30 s, annealing at 52°C for 30 s, elongation at 72°C for 2 min), terminal extension at 72°C for 5 min, and cooling at 4°C. Purification and sequencing of the PCR products were performed with the same settings as above using the bacterial primers 27F and 1492R. GenBank accession numbers of obtained thecofilosean and bacterial sequences are given in **Supplementary Table S1**.

### Probe Design and Fluorescence *In Situ* Hybridization

An alignment of the obtained bacterial sequences and their top ten hits from a BLAST search (Basic Local Alignment Tool) and close relatives was assembled in SeaView (Gouy et al., 2010). The alignment was screened for short sequence segments with lengths of 16–20 bases in which the respective bacteria group differed from all the others. The short sequence segments were searched against the SILVA database (Pruesse et al., 2007) using the Probe Match and Evaluation Tool TestProbe 3.0^1^ until sequence segments as specific as possible to the desired bacteria group were found. Short sequences fulfilling these requirements were tested with the OligoEvaluator tool (Sigma-Aldrich)^2^ to find sequences without secondary structures to avoid self-binding or folding of the FISH probes. Reverse compliment FISH probes were ordered from biomers.net GmbH^3^ as oligonucleotides with either Cy3 or FITC as 5′-modification. The resulting probes and their numbers of matches in the SILVA database are displayed in **Supplementary Table S2**.

Between 20 and 200 µl of cultures were air-dried on 0.1% gelatine-coated glass slides and dehydrated in an increasing ethanol series (50, 80, and 96%) for 3 min each and finally air-dried again. Subsequently, 100 µl hybridization buffer (18 µl 5M NaCl solution, 2 µl 1M TrisHCl solution, 35 µl formamide, 45 µl water, 0.1 µl 10% SDS) was mixed with 12.5 µl (100 µM) of the general bacteria probe EUB338 (Amann and Fuchs, 2008) tagged with FITC and a specific probe tagged with Cy3 (**Supplementary Table S2**), applied to the samples, and incubated for 90 min (48°C in a moist chamber). Subsequently, samples were incubated in preheated washing buffer (700 µl 5M NaCl solution, 1,000 µl 1M TrisHCl solution, 50 ml water, and 50 µl 10% SDS) for 25 min at 48°C. Air-dried slides were stained with DAPI for 10 min, washed, air-dried, and subsequently mounted with 85% glycerol and 15% phosphate-buffered saline (PBS) [after Manz et al. (1992); Hugenholtz et al. (2001); Hess et al. (2016)]. The general probes EUB338, ALF968, BET42a, and GAM42a (Amann and Fuchs, 2008) and DAPI staining were used as controls before taxon-specific probes were applied. Pictures of the processed cells were taken with a Nikon digital sight DS-U2 camera (program: NIS-Elements V4.13.04) with a Nikon Eclipse 90i upright microscope (up to 600× magnification).

### Phylogenetic Analyses

All partial sequences were checked for sequencing errors in Chromas (V2.6.6, Technelysium Pty. Ltd., Australia) before they were assembled into one sequence contig using SeaView [V4.6, (Gouy et al., 2010)]. For the thecofilosean alignment, representative sequences of major thecofilosean subphyla were added to the sequences of the Thecofilosea from this study. Ten selected Imbricatea sequences were used as outgroup. The probes LE1_10_Gam, Ft_Chryseo, 5_Sedimini did not result in positive staining when applied to cells of the respective cultures. Therefore, only bacteria successfully stained with the probes LE1_3B_3C_Gam, 3_5_6_Thec_Gam, WM_Legio, or RC_Rick, respectively, have been used for phylogenetic analyses. Accordingly, two separate phylogenetic analyses have been performed for endosymbiotic bacteria belonging to the Legionellales (Gammaproteobacteria) and Rickettsiales (Alphaproteobacteria).

To create the datasets for phylogenetic analyses, the top ten hits of each verified endosymbiotic bacterium were obtained from the NCBI GenBank database (last date of accession: July 26^th^, 2019) by using the blastn search algorithm (blastn 2.3.0) with default parameters. For the Legionellales dataset, all available *Legionella* sequences were downloaded from the SILVA database (https://www.arb-silva.de/) and ordered by species names. Sequences of undescribed *Legionella* spp. were removed, and only one sequence of every described species was maintained. Based on the overview of the order Legionellales by Duron et al. (2018), selected sequences of the major subphyla were added to the dataset, including sequences of ten *Diplorickettsia*, ten *Rickettsiella*, ten *Aquicella*, ten *Coxiella*, two “*Ca*. Berkiella”, “*Ca*. Nucleophilum amoebae” and “*Ca*. Occultobacter vannellae”. 13 selected *Pseudomonas* sequences were used as outgroup. For the phylogenetic analyses of the Rickettsiales sequences from Hess et al. (2016) were obtained and additional “*Candidatus* Megaira” sequences from Schrallhammer et al. (2013) were added. Sequences were aligned in MAFFT using the L-INS-i algorithm (Katoh and Toh, 2010) and ambiguously aligned sequence segments were manually cut using SeaView. The model GTR + I + G was used and maximum likelihood (ML) phylogenetic trees were constructed using RAxML (Randomized Axelerated Maximum Likelihood, Version 8, (Stamatakis, 2014)). The best scoring tree was used to report the confidence values as percentages obtained through 200 non-parametric bootstraps under the GTRCAT model.

## Results

### Endosymbiont Detection and Phylogeny

**Figure 1:**
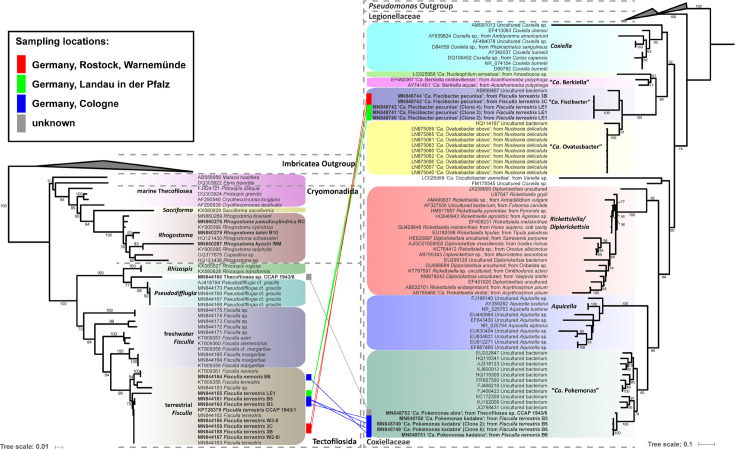
Phylogenetic trees of thecofilosean amoebae and their respective endosymbionts of the Coxiellaceae (Legionellales, Gammaproteobacteria). Lines connect the thecofilosean hosts and their respective endosymbionts; line color indicates sampling locations (see legend). Only confidence values >50 are displayed.

**Figure 2:**
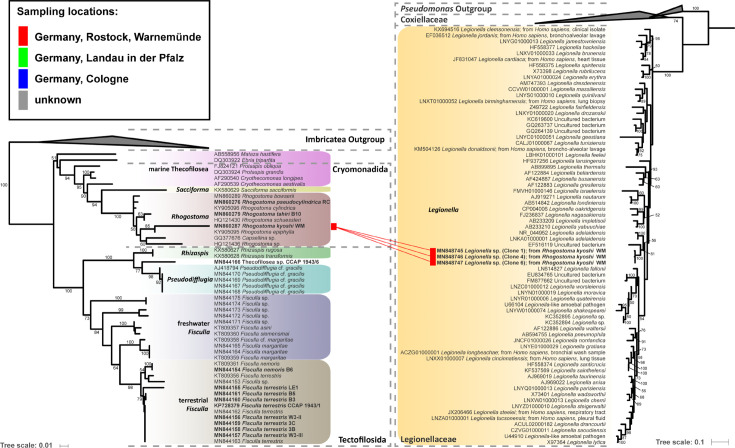
Phylogenetic trees of thecofilosean amoebae and their respective endosymbionts of the Legionellaceae (Legionellales, Gammaproteobacteria). Lines connect the thecofilosean hosts and their respective endosymbionts; line color indicates sampling locations (see legend). Only confidence values >50 are displayed.

**Figure 3:**
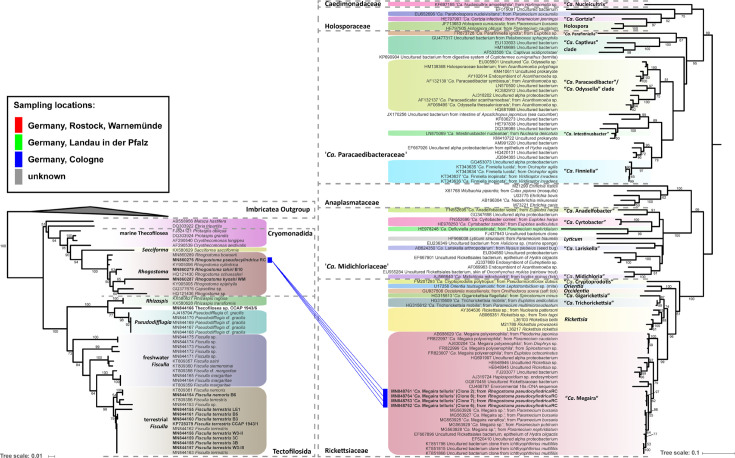
Phylogenetic trees of thecofilosean amoebae and their respective endosymbionts of the Rickettsiales (Alphaproteobacteria). Lines connect the thecofilosean hosts and their respective endosymbionts; line color indicates sampling locations (see legend). The tree contains no outgroup, but was rooted to resemble the tree from Hess et al. (2016). Only confidence values >50 are displayed.

Eleven thecofilosean cultures from environmental samples and two from the CCAP culture collection were screened for endosymbiotic bacteria (**Supplementary Table S1**). Eight of the 13 thecofilosean amoebae cultures contained Legionellales endosymbionts confirmed by sequencing and fluorescence *in situ* hybridization (FISH). From these, we obtained in total 13 Legionellales 16S sequences, three of which could be assigned to a novel *Legionella* isolate. For the remaining sequences the novel candidate species “*Ca*. Fiscibacter pecunius”, “*Ca*. Pokemonas kadabra”, and “*Ca*. Pokemonas abra” (**Figures 1**, **2** **; Supplementary Material**) were erected. “*Ca*. Fiscibacter pecunius” was detected in three different strains of *Fisculla terrestris* (LE1, 3B, 3C), “*Ca*. Pokemonas kadabra” in two strains of *Fisculla terrestris* (B3, B5) and one strain of *Fisculla nemoris* (B6), and “*Ca*. Pokemonas abra” in one strain of Thecofilosea sp. (CCAP 1943/6) (**Figure 1**). The *Legionella* sp. was found in a strain of *Rhogostoma kyoshi* (WM) (**Figure 2**). Only one non-Legionellales endosymbiont was detected in a strain of *Rhogostoma pseudocylindrica* (RC), and based on 16S rDNA phylogeny it could be assigned to the genus “*Ca.* Megaira” in Rickettsiales (**Figure 3**).

“*Ca*. Fiscibacter pecunius”, “*Ca*. Pokemonas abra” and “*Ca*. Pokemonas kadabra” formed distinct clades within the Coxiellaceae (**Figure 1**). “*Ca*. Fiscibacter” branches are highly supported close to “*Ca*. Ovatusbacter abovo” which was described by Dirren and Posch (2016) as a member of Gammaproteobacteria. Dirren and Posch (2016) already noticed high sequence similarities of “*Ca*. Ovatusbacter abovo” to “*Ca*. Berkiella”, but did not assign a family. In our analysis, they represent a clade within the family Coxiellaceae. “*Ca*. Pokemonas” represents a highly supported sister group to *Aquicella*. The closest described relative to the endosymbiotic *Legionella* sp. (Legionellaceae, Legionellales) was *Legionella adelaidensis* with a bootstrap value of 56% (**Figure 2**).

**Figure 4:**
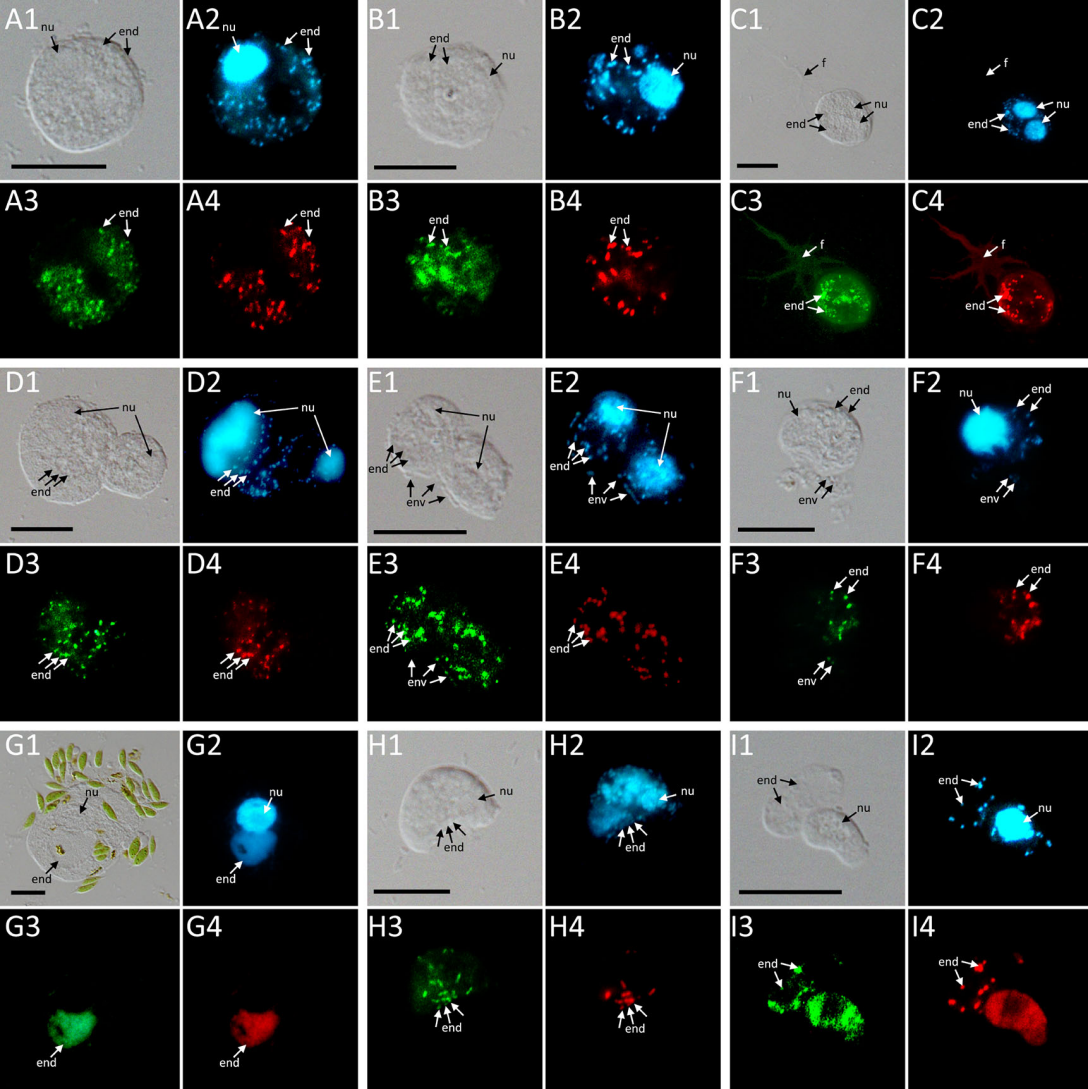
FISH pictures. **(A–C)**: Strains containing “*Ca*. Fiscibacter pecunius”. **(A)** *F*. *terrestris* (strain LE1); **(B)** *F*. *terrestris* (strain 3B); **(C)** *F*. *terrestris* (strain 3C). **(D–F)**: Strains containing “*Ca*. Pokemonas kadabra”. **(D)** *F*. *terrestris* (strain B3); **(E)** *F*. *terrestris* (strain B5); **(F)** *F*. *nemoris* (strain B6). **(G)** Thecofilosea sp. (strain CCAP 1943/6) containing “*Ca*. Pokemonas abra”. **(H)** *Rhogostoma kyoshi* (strain WM) containing *Legionella* sp. **(I)** *Rhogostoma pseudocylindrica* (strain RC) containing “*Ca*. Megaira telluris”. 1: Difference interference contrast (DIC); 2: DAPI staining; 3: staining with general bacteria probe EUB338; 4: staining with specific probe (see **Supplementary Table S2**). Scale bars indicate 10 μm. Nu, nucleus; f, filopodia; env, environmental bacteria; end, endosymbiotic bacteria.

Phylogenetic analyses of the hosts and endosymbionts show no clear co-evolutionary pattern, indicating a promiscuous dispersal of endosymbiotic bacteria in the Thecofilosea (**Figure 1**). No endosymbionts were detected in four of the 13 screened amoeba strains, indicating that the identified endosymbiotic bacteria in Thecofilosea are facultative endosymbionts for the host. In all but one thecofilosean amoeba, the endosymbionts were dispersed throughout the whole cell body, except in the nucleus and the filopodia (**Figure 4**). Only in one host strain (Thecofilosea sp. 1943/6), the Legionellales endosymbionts were concentrated in one large vacuole (**Figure 4G**). The vacuole was present in all individuals and usually larger than the hosts’ nucleus. In all strains, the endosymbionts were transmitted vertically by cell division (see a dividing cell of *Fisculla terrestris* in **Figure 4E**). Extracellular transmission of bacteria was not observed.

For the first time, long-term stable cultures consisting of the *Fisculla* spp., their bacterial symbiont (Legionellales spp.), and the eukaryotic prey (*S. cerevisisae*) were established (see *Material and Methods*). These cultures can be specified as gnotobiotic, *i.e*. all organisms in the culture (host, endosymbiont, and food organisms) are known. All nine established *Fisculla* spp. cultures were free of environmental bacteria and stable over a year, the whole runtime of the project.

## Discussion

### Legionellales Proliferate in Amoebal Hosts of Widespread Evolutionary Origin

**Figure 5:**
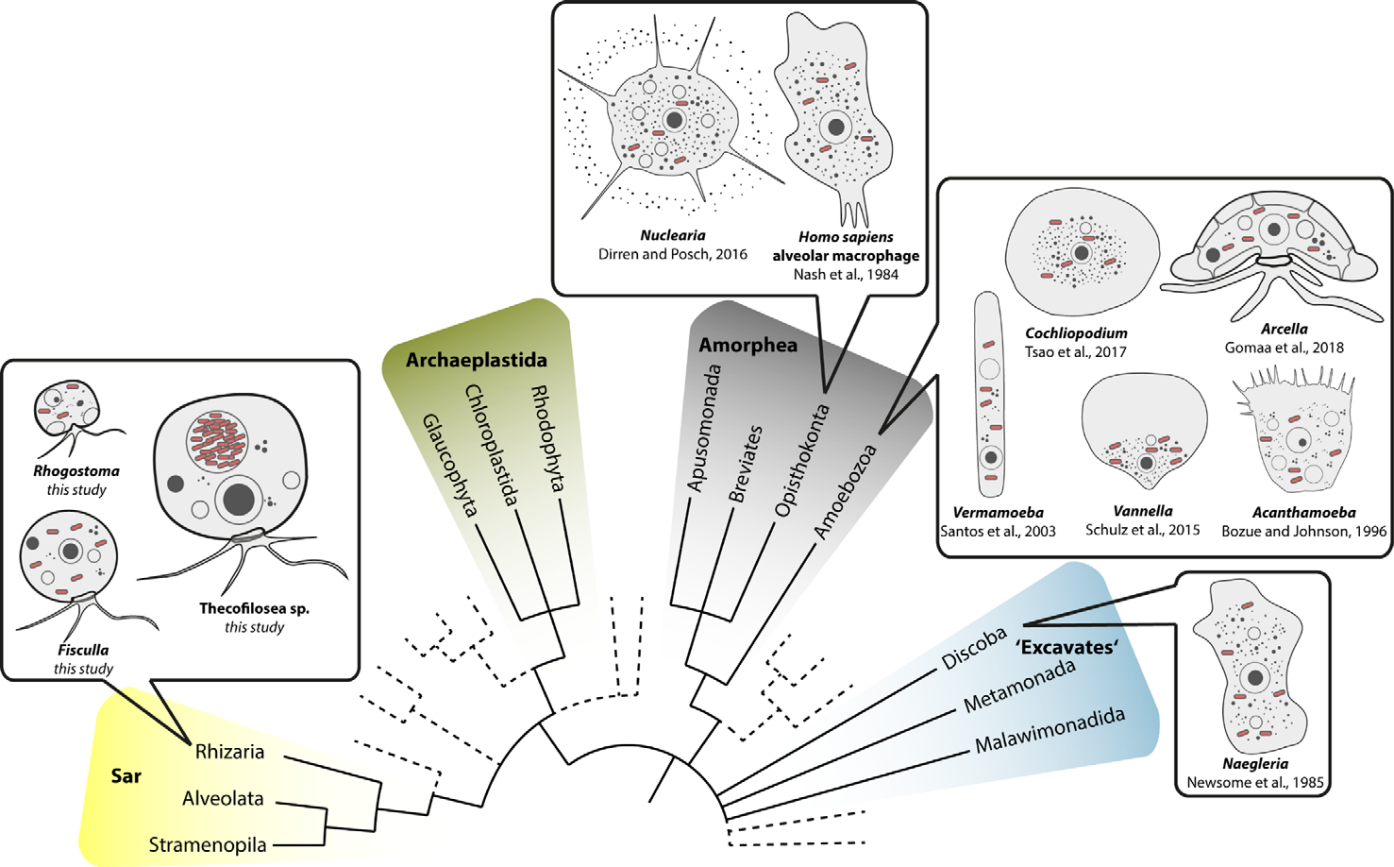
Overview of eukaryotic diversity based on (Burki et al., 2019). Former eukaryotic supergroups are highlighted, Opisthokonta and Amoebozoa are combined into Amorphea, and novel or minor groups are reduced. Depicted is the known diversity of amoebal Legionellales hosts. Note that prior to this study most Legionellales were found in amorphean amoebae and only one excavate taxon.

Our data on Thecofilosea clearly show that amoebae in the phylum Cercozoa accommodate a variety of bacterial endosymbionts in Legionellales. The fact that Legionellales are not restricted to Amorphea has important applied implications because our data expand the potential host range of Legionellales to the evolutionary very distantly related Rhizaria (**Figure 5**). From an evolutionary perspective, it is crucial to trace back Legionellales’ diversity to their respective hosts to understand and predict the bacterial adaptations to the immune system of their respective hosts, a prerequisite for the development of antimicrobial measures/treatments (Molmeret et al., 2005; Duron et al., 2018; Gomez-Valero and Buchrieser, 2019; Mondino et al., 2020).

Despite numerous publications on diverse bacterial endosymbionts of protists, there are few reports of Legionellales. The ciliate protist *Euplotes* is commonly used as a model for endosymbiosis research; however, none of the yet determined dozens of different endosymbionts of *Euplotes* belonged to Legionellales (Boscaro et al., 2019). *Euplotes* differs in morphology from the majority of known potential Legionellales hosts which are amoeboid (**Figure 5**). It seems that the evolutionary origin of the potential host does not indicate the susceptibility for infection by Legionellales since potential hosts belong to rather unrelated eukaryotes. Instead, it is striking that amoebae, a morphological term and not an evolutionary lineage, function exceptionally often as hosts for Legionellales. Even in humans, the infected cells are much alike an amoeba since the infected alveolar macrophages of the human immune system are surface-attached grazing cells (Duron et al., 2018).

Legionellales are long known to be associated with biofilms, *i.e.* they are more frequent on submerged surfaces than in water columns (Rogers et al., 1994; Abu Khweek and Amer, 2018). Our results consolidate the comprehension of why and how Legionellales are associated with biofilms. By far most amoebae swim only occasionally; most often they are substrate-attached, accordingly, amoebae are among the most abundant grazers on biofilms, where they engulf prey, including whole batches of bacteria (Bonkowski, 2019). Other protist groups, like flagellates and ciliates often preferentially predate freely swimming prey (Verni and Gualtieri, 1997; Boenigk and Arndt, 2002), and as we hypothesize that is why they come in less contact with substrate attached Legionellales and are thus only rarely or never infected.

To our knowledge, it is still questionable whether Legionellales replicate under natural conditions without a eukaryotic host (Fields et al., 2002). It was shown that a lack of amoebae prevents growth of the substrate-attached *Legionella pneumophila* (Declerck et al., 2009; Declerck, 2010). *Legionella* can be grown axenically in the lab, but for this, exact conditions have to be met. It was proposed that, even if such conditions are met environmentally, other bacteria would quickly outcompete potentially freely replicating Legionellales (Fields et al., 2002). Although some amoebal hosts are lysed by their endosymbionts (La Scola et al., 2004) our and other studies did not find any evidence for lysis of the host cell after replication of the Legionellales indicating that Legionellales may form stable and long term symbiosis with their host (Gomaa et al., 2018). Subsequently, this means that not necessarily the biofilms, but the amoebae within, form a reservoir for Legionellales.

While the pathogenic potential of the novel described “*Ca*. Pokemonas” and “*Ca*. Fiscibacter pecunius” cannot be inferred by our data, the found *Legionella* sp. is closely related to *Legionella adelaidensis* that belongs to a group with many waterborne human pathogenic species. The found *Legionella* sp. was detected in a strain of *Rhogostoma kyoshi* that was obtained from biological soil crusts, *i.e.* surface soil (Khanipour Roshan et al., 2021). *Rhogostoma* represents a genus of thecofilosean amoebae that is especially abundant in soils (Degrune et al., 2019; Fiore-Donno et al., 2019; Öztoprak et al., 2020), wastewater treatment plants (Matsunaga et al., 2014; Remmas et al., 2016a; Remmas et al., 2016b; Öztoprak et al., 2020), and water filters (Domingo Fernandez, 2019), and their potential as a reservoir for Legionellales requires further investigation.

The nature of the relationship between the reported endosymbionts and their hosts, whether being mutualistic or parasitic, was not investigated in this study, but the gnotobiotic cultivation method of *Fisculla* spp. facilitates future genomic and transcriptomic studies, helping to answer these open research questions.

## Data Availability Statement

The datasets presented in this study can be found in online repositories. The names of the repository/repositories and accession number(s) can be found in the article/**Supplementary Material**.

## Author Contributions

KD conceived the project. KD and MB administrated the project. MS and KD conducted the amplifications, sequencing, FISH staining, and phylogenetic analyses. MS wrote the initial manuscript, KD and MB revised it. All authors contributed to the article and approved the submitted version.

## Conflict of Interest

The authors declare that the research was conducted in the absence of any commercial or financial relationships that could be construed as a potential conflict of interest.

## Footnotes

1
https://www.arb-silva.de/search/testprobe/

2
http://www.oligoevaluator.com/

3
https://www.biomers.net/
