# Supplementary material for: Novel Endosymbionts in Rhizarian Amoebae Imply Universal Infection of Unrelated Free-Living Amoebae by Legionellales
Source: Front Cell Infect Microbiol. 2021 Mar 8;11:642216. doi: 10.3389/fcimb.2021.642216 (PMC7982676; doi:10.3389/fcimb.2021.642216)
Supplement: Supplementary file 1 [file DataSheet_1.pdf]

frontiers

Supplementary Material

1 Extended Material and Methods

Supplementary Table S1: Thecofilosean strains examined in this study. Included are determined species, information about sampling location, isolation date and culture conditions.

Amoeba species	Strain	CCAP ref.	GenBank Accession number	Sampling location	Habitat type	Coordinates	Isolation date	Culture medium	Food source in culture	Endosymbiont	Endosymbiont accession number
Fisculla nemoris	B6	1922/1	MN844154	Germany, Cologne	leaves	50.927186, 6.935997	December 2018	Waris-H + Si	*Saccharomyces cerevisiae*	"*Ca*. Pokemonas kadabra"	MN848751
Fisculla terrestris	CCAP 1943/1	1943/1	KP728379	Germany, Berlin, Müncheberg	soil	52.517906, 14.122767	-	Waris-H + Si	*Saccharomyces cerevisiae*	-	-
Fisculla terrestris	3B	1922/2	MN844158	Germany, Rostock, Warnemünde	soil crust	54.180267, 12.080450	February 2019	Waris-H + Si	*Saccharomyces cerevisiae*	"*Ca*. Fiscibacter pecunius"	MN848744
Fisculla terrestris	3C	1922/3	MN844159	Germany, Rostock, Warnemünde	soil crust	54.180267, 12.080450	February 2019	Waris-H + Si	*Saccharomyces cerevisiae*	"*Ca*. Fiscibacter pecunius"	MN848743
Fisculla terrestris	B3	1922/4	MN844160	Germany, Cologne	leaves	50.927186, 6.935997	December 2018	Waris-H + Si	*Saccharomyces cerevisiae*	"*Ca*. Pokemonas kadabra"	MN848750
Fisculla terrestris	B5	1922/5	MN844161	Germany, Cologne	leaves	50.927186, 6.935997	December 2018	Waris-H + Si	*Saccharomyces cerevisiae*	"*Ca*. Pokemonas kadabra"	MN848748<br>MN848749

Fisculla terrestris	**LE1**	**1922/6**	**MN844155**	**Germany, Landau in der Pfalz**	**litter**	**49.205444, 8.087611**	**December 2018**	**Waris-H + Si**	***Saccharomyces cerevisiae***	**"*Ca.* Fiscibacter pecunius"**	**MN848740**<br>**MN848741**<br>**MN848742**
Fisculla terrestris	**W3-II**	**1922/7**	**MN844156**	**Austria, Vienna**	**lichen**	**48.207667, 16.366056**	**February 2019**	**Waris-H + Si**	***Saccharomyces cerevisiae***	**-**	**-**
Fisculla terrestris	**W3-III**	**1922/8**	**MN844157**	**Austria, Vienna**	**lichen**	**48.207667, 16.366056**	**February 2019**	**Waris-H + Si**	***Saccharomyces cerevisiae***	**-**	**-**
Rhogostoma pseudocylindrica	**RC**	**1966/5**	**MN860276**	**Germany, Cologne**	**soil**	**50.927186, 6.935997**	**October 2017**	**WG**	***Escherichia coli***	**"*Ca.* Megaira telluris"**	**MN848761**<br>**MN848762**<br>**MN848763**<br>**MN848764**
Rhogostoma tahiri	**B10**	**1966/6**	**MN860279**	**Germany, Cologne**	**leaves**	**50.927186, 6.935997**	**December 2018**	**WG**	***Escherichia coli***	**-**	**-**
Rhogostoma kyoshi	**WM**	**1966/8**	**MN860287**	**Germany, Rostock, Warnemünde**	**soil crust**	**54.180267, 12.080450**	**December 2018**	**WG**	***Escherichia coli***	***Legionella* sp.**	**MN848745**<br>**MN848746**<br>**MN848747**
**Thecofilosea sp.**	**CCAP 1943/6**	**1943/6**	**MN844166**	**-**	**freshwater**	**-**	**-**	**WC**	***Characium* sp., *Nitzschia communis* (CCAC 5737B), *Nitzschia amphibia* (CCAC 5733B)**	**"*Ca.* Pokemonas abra"**	**MN848752**

Supplementary Table S2: FISH probes designed in this study. Probes in bold resulted in positive staining. *Number of hits in the SILVA database using TestProbe (last accession: August 12th, 2019).

**Probe name**	**Sequence (5‘-3‘)**	**Target bacteria**	**Host strains**	**Hits***
**LE1_3B_3C_Gam**	**GCCCTTGCGAGCCTGTTA**	**“*Ca*. Fiscibacter pecunius”**	***Fisculla terrestris* LE1**<br><br>***Fisculla terrestris* 3B**<br><br>***Fisculla terrestris* 3C**	**0**
**3_5_6_Thec_Gam**	**TCTCTCAGCGCTCGGTTTA**	**“*Ca*. Pokemonas abra”**<br><br>**“*Ca*. Pokemonas kadabra”**	**Thecofilosea sp. CCAP 1943/6**<br><br>***Fisculla terrestris* B3**<br><br>***Fisculla terrestris* B5**<br><br>***Fisculla nemoris* B6**	**0**
**WM_Legio**	**CCTAAGTACCCTCCTCCCCA**	***Legionella* sp.**	***Rhogostoma kyoshi* WM**	**4**
**RC_Rick**	**CTGAAGCAAGCTCCAAAATT**	**“*Ca*. Megaira telluris”**	***Rhogostoma pseudocylindrica* RC**	**0**
LE1_10_Gam	TCCCAACCACCTACACACG	*Undescribed Gammaproteobacterium*	*Rhogostoma tahiri* B10<br><br>*Fisculla terrestris* LE1	3
Ft_Chryseo	GCGCCGCTCTCAAGTATC	*Chryseobacterium*	*Fisculla terrestris* CCAP 1943/1	12
5_Sedimini	AGCCGCACACCCATCAAT	*Sediminibacterium*	*Fisculla terrestris* B5	37

2 Taxonomic Appendix

Most of the recently described Legionellales have been characterized solely by their 16S rRNA gene sequences (e.g. Schulz *et al*., 2015; Dirren and Posch, 2016; Mehari *et al*., 2016), prompting us to use this gene for our phylogenetic analysis and taxonomic placements. We acknowledge that the low polymorphism of this gene often results in partially unresolved phylogenetic relationships which impede the identification of exact species relatedness, which is also reflected in our phylogenetic analysis by the low support of Legionelalles monophyly and the grouping of “*Ca*. Berkiella” and *Coxiella* (Duron et al., 2018). We also acknowledge that future phylogenies with larger sequence datasets may make major taxonomic changes within Gammaproteobacteria and the Legionellales necessary (Mehari et al., 2016; Hugoson et al., 2019). Also, axenic cultures cannot be established from endosymbionts, which is a prerequisite of The International Code of Nomenclature of Prokaryotes (ICNP) when describing prokaryotes. Accordingly, we describe provisional taxa with the “Candidatus”

status for incompletely described prokaryotes as proposed by Murray and Stackebrandt (1995). Host strains are available from the Culture Collection of Algae and Protozoa (CCAP).

We establish the novel candidate species "*Ca.* Fiscibacter pecunius" (Coxiellaceae, Legionellales, Gammaproteobacteria), the novel candidate genus "*Ca.* Pokemonas" (Coxiellaceae, Legionellales, Gammaproteobacteria) including two novel candidate species, and a novel candidate species belonging to the genus "*Ca.* Megaira" (Rickettsiales, Alphaproteobacteria):

"*Candidatus* Fiscibacter pecunius" [(Coxiellaceae, Gammaproteobacteria) NC; NA; n.a.; nucleic acid sequence (NAS) (GenBank numbers MN848740, MN848741, MN848742, MN848743, MN848744); oligonucleotide sequence complementary to unique region of 16S rRNA 5'-GCCCTTGCGAGCCTGTTA-3' (Probe LE1_3B_3C_Gam); S (*Fisculla terrestris* strains LE1, 3B, 3C {Cercozoa}, cytoplasm); Aer.; M]. Etymology: fiscus, m (=money bag) [Latin], bacter, m (=rod) [New Latin]; pecunia, f (=money) [Latin], -us, masculine suffix [Latin]. The genus name "Fiscibacter" refers to the host organisms (genus *Fisculla*) the bacteria were found in. The species name "pecunius" refers to the intracellular occurrence of the bacteria, like coins in a purse. Additional information: Cells scattered throughout the cytoplasm of *Fisculla terrestris* cells; phylogenetically defined as a well-supported clade within the Coxiellaceae, containing the sequences of "*Ca.* Fiscibacter pecunius LE1", "*Ca.* Fiscibacter pecunius 3B", "*Ca.* Fiscibacter pecunius 3C". Reference strain: "*Ca.* Fiscibacter pecunius 3C" (inhabiting *Fisculla terrestris* strain 3C). Locality of reference strain: Soil crust sample from a sand beach near the Baltic Sea, Warnemünde, Rostock, Germany; 54.180267, 12.080450.

"*Candidatus* Pokemonas" [(Coxiellaceae, Gammaproteobacteria) NC; NA; n.a.; nucleic acid sequence (NAS) (GenBank numbers MN848748, MN848749, MN848750, MN848751, MN848752), oligonucleotide sequence complementary to unique region of 16S rRNA 5'-TCTCTCAGCGCTCGGTTTA-3' (Probe 3_5_6_Thec_Gam); S (*Fisculla terrestris* strains B3, B5, *Fisculla nemoris* strain B6, Thecofilosea sp. strain CCAP 1943/6 {Cercozoa}, cytoplasm); Aer.; M]. Etymology: Pokémon (ポケットモンスター Poketto Monsutā, =Pocket Monsters) [Japanese], monas, f (μονάς, =single unit) [Greek]. The name "Pokemonas" alludes to the video game franchise "Pokémon" in which monsters can be caught in balls and carried in the pocket. This refers to the intracellular occurrence of the bacteria. Additional information: Cells colonizing the cytoplasm of thecofilosean amoebae (Cercozoa, Rhizaria); phylogenetically defined as a well-supported clade within the Coxiellaceae, containing sequences of the candidate species "*Ca.* Pokemonas abra" and "*Ca.* Pokemonas kadabra". Proposed type candidate species: "*Ca.* Pokemonas abra". Taxa currently included: "*Ca.* Pokemonas abra", "*Ca.* Pokemonas kadabra".

"*Candidatus* Pokemonas abra" [(Coxiellaceae, Gammaproteobacteria) NC; NA; n.a.; nucleic acid sequence (NAS) (GenBank number MN848752); S (Thecofilosea sp. strain CCAP 1943/6 {Cercozoa}, cytoplasm); Aer.; M]. Etymology: The name "abra" derives from the Pokémon called "Abra", as an allusion to the incantation "Abrakadraba Simsalabim". Additional information: Cells condensed in a

large vacuole in the cytoplasm of Thecofilosea sp. cells; phylogenetically defined to contain the sequences of "*Ca.* Pokemonas abra CCAP 1943/6", but not those of "*Ca.* Pokemonas kadabra". Reference strain: "*Candidatus* Pokemonas abra CCAP 1943/6" (inhabiting Thecofilosea sp. strain CCAP 1943/6).

"*Candidatus* Pokemonas kadabra" [(Coxiellaceae, Gammaproteobacteria) NC; NA; n.a.; nucleic acid sequence (NAS) (GenBank numbers MN848748, MN848749, MN848750, MN848751); S (*Fisculla terrestris* strains B3, B5, *Fisculla nemoris* strain B6 {Cercozoa}, cytoplasm); Aer.; M]. Etymology: The species name "kadabra" derives from the Pokémon called "Kadabra", as an allusion to the incantation "Abrakadraba Simsalabim". Additional information: Cells scattered throughout the cytoplasm of *F. terrestris* or *F. nemoris* cells; phylogenetically defined to contain the sequences of "*Ca.* Pokemonas kadabra B3", "*Ca.* Pokemonas kadabra B5", "*Ca.* Pokemonas kadabra B6", but not those of "*Ca.* Pokemonas abra CCAP 1943/6". Reference strain: "*Candidatus* Pokemonas kadabra B3" (inhabiting *Fisculla terrestris* strain B3). Locality of reference strain: Leaf sample from the courtyard of the Biocenter, Cologne, Germany; 50.927186, 6.935997.

"*Candidatus* Megaira telluris" [(Rickettsiales, Alphaproteobacteria) not cultivated (NC); not applicable (NA); n.a.; nucleic acid sequence (NAS) (GenBank numbers MN848761, MN848762, MN848763, MN848764); oligonucleotide sequence complementary to unique region of 16S rRNA 5'-CTGAAGCAAGCTCCAAAATT-3' (Probe Rc_Rick); symbiotic (S) (*Rhogostoma pseudocylindrica* strain RC {Cercozoa}); aerobic (Aer.); mesophilic (M)]. Etymology: tellus f (=ground, earth) [Latin], genitive telluris. The species name "telluris" refers to the host organism that was obtained from soil. Additional information: Cells scattered throughout the cytoplasm of *Rhogostoma pseudocylindrica* cells; phylogenetically defined as a clade within the provisional genus "*Candidatus* Megaira". Reference strain: "*Candidatus* Megaira telluris RC" (inhabiting *Rhogostoma pseudocylindrica* strain RC). Locality of reference strain: Soil sample from the courtyard of the Biocenter, Cologne, Germany; 50.927186, 6.935997.

References

Dirren, S., and Posch, T. (2016). Promiscuous and specific bacterial symbiont acquisition in the amoeboid genus Nuclearia (Opisthokonta). *FEMS Microbiol. Ecol.* 92, 1–16. doi:10.1093/femsec/fiw105.

Duron, O., Doublet, P., Vavre, F., and Bouchon, D. (2018). The Importance of Revisiting Legionellales Diversity. *Trends Parasitol.* 34, 1027–1037. doi:10.1016/j.pt.2018.09.008.

Hugoson, E., Ammunét, T., and Guy, L. (2019). Host-adaptation in Legionellales is 2.4 Gya, coincident with eukaryogenesis. *bioRxiv*, 1–41. doi:10.1101/852004.

Mehari, Y. T., Hayes, B. J., Redding, K. S., Mariappan, P. V. G., Gunderson, J. H., Farone, A. L., et

al. (2016). Description of ‘Candidatus Berkiella aquae’ and ‘Candidatus Berkiella cookevillensis’, two intranuclear bacteria of freshwater amoebae. *Int. J. Syst. Evol. Microbiol.* 66, 536–541. doi:10.1099/ijsem.0.000750.

Schulz, F., Tyml, T., Pizzetti, I., Dyková, I., Fazi, S., Kostka, M., et al. (2015). Marine amoebae with cytoplasmic and perinuclear symbionts deeply branching in the Gammaproteobacteria. *Sci. Rep.* 5, 1–10. doi:10.1038/srep13381.
